# A survey on occupational injuries and related factors among emergency patients of Mashhad teaching hospitals over a year

**DOI:** 10.5249/jivr.v16i1.1902

**Published:** 2024-01

**Authors:** Hosein Zakeri, Hasan Jahed Taherani, Lahya Afshari Saleh

**Affiliations:** ^*a*^ Department of Emergency Medicine, Faculty of Medicine, Mashhad University of Medical Sciences, Mashhad, Iran.; ^*b*^ Department of Occupational Medicine, Faculty of Medicine, Mashhad University of Medical Sciences, Mashhad, Iran.; ^*c*^ Department of Occupational Medicine, Division of Sleep Medicine, Psychiatry and Behavioral Sciences Research Center, Mashhad University of Medical Sciences, Mashhad, Iran.

**Keywords:** Occupational injury, Work-related trauma, Emergency room

## Abstract

**Background:**

Occupational injuries (OI) are a - significant source of social and economic costs. They can cause prolonged absences from work, loss of limb, and worker disability, among other negative consequences. In light of this, the present study aimed to evaluate the factors associated with OI in trauma centers affiliated with Mashhad University of Medical Sciences.

**Methods:**

This cross-sectional study was carried out among patients admitted to the trauma ward of four teaching hospitals in Mashhad during 2019-2020. Patients’ data was collected in a checklist and trauma severity was assessed based on injury severity score. The Epworth questionnaire was completed for patients. Data was analyzed by SPSS 20.0.

**Results:**

Out of a total of 13660 patients who were referred to the emergency department of four hospitals of Mashhad University of Medical Sciences, 683 (5.15%) individuals suffered occupational trauma and entered the study. The mean (±SD) age and work experience of the participants were 34 (±10) and 10 (±9) respectively. Most of the participants were in the age group of 20-40 years. the most common mechanism of trauma was falling (p=0.00) and the most injury frequency was among manual working (p=0.01). There was not a significant association between trauma severity and demographic characteristics. Those who had falling or electric accidents had more severe trauma compared to other injured patients. (p=0.00).

**Conclusions:**

In general, the most burden of occupational accidents and in fact, the most serious injuries were related to men and manual workers. Also, most of the injuries happened through falls and cuts. The results of this study showed the need for serious policies to reduce occupational injuries.

## Introduction

According to a report by the World Health Organization, there are approximately 120 million occupational accidents (OA) worldwide, annually. These accidents lead to more than 2 million workers suffering from disabilities. About 170 million workdays are wasted because of occupational accidents annually, and at least 4 people get injured every second. Many people spend more than one-third of their adolescence life at workplaces and during this time, they are faced with different occupational hazards. Occupational injuries cause significant social and economic costs.^1^ Despite attempts to improve occupational health and safety, many occupational accidents cause serious injuries and death every year.^2^

OA not only causes suffering for a person and his/her family but also increases general costs by lost efficiency and increased use of medical care and welfare services. Society-related costs were estimated between 2-14 percent in studies in different countries.^3^

Occupational injury is any type of injury that is related to a person's job needs or desires. Occupational injury (OI) includes a significant percentage of emergency department patients as well.^4^ Identifying the most possible risk factors in the incidence of occupational injuries at a specific workplace, could be considered as a base for prevention and reducing the severity of the accidents. Also, improving the environment of workplaces to prevent accidents and work-related diseases is one of the major tasks of industrial managers and healthcare staff.^5^

In previous studies, multiple factors were correlated with occupational trauma, such as male gender, age, job type (agricultural jobs, technical and construction jobs, high-stress jobs like carrying heavy loads, awkward working postures, environmental stress, working under pressure), workers’ health-related variables like lack of physical activity, smoking, drinking alcohol and obesity.^6^

In another study on occupational injuries, the severity of injuries was added to the above-mentioned collection, which has an important implication on emergency services, hospital bed occupancy, and the workload of ICU.^7^

The frequency of occupational accidents in developing countries is significantly higher compared to that of developed countries. This is primarily due to the fact that developing countries account for 60% of the global workforce, and the job security situation in developed countries is better than in developing countries. According to the statistical data released by the International Labor Organization, the number of deaths in developed countries is on average 4 per hundred thousand people, and in developing countries, it is over 10 per hundred thousand people.^8,9^ The incidence of occupational trauma was reported1.37% among Korean workers in 2006^10^ 69.93% among Chinese migrant workers in 2017 ^11^ and 42.2% among Argentinean farmers in 2015.^12^

In Iran, the rate of work-related accidents among individuals insured by the Social Security Organization throughout the country was found to be 0.3 between 1980-1984, and this rate has increased by 0.3% annually. A study conducted in the Yazd province showed that the frequency of such incidents was 1.56%, whereas in 2008, it was reported to be 0.25% in the Tehran province.^13^

This study was conducted in order to investigate the incidence of occupational traumas and their associated factors in four emergency centers affiliated with Mashhad University of Medical Sciences.

## Methods 

The current study employed an observational cross-sectional design and examined patients with occupational injuries who were admitted or referred to the Emargency Room during the period of 2019-2020. Occupational injuries are - defined as any injury that arises from a person's occupational needs or desires.^4^ The diagnosis of occupational injuries was based on a comprehensive history taken from the trauma patient taken by an emergency medicine specialist. Participants who did not have complete information recorded or did not complete the questionnaires were excluded from the study.

The study instrument comprised three components, namely:

A. A checklist containing demographic and job description details, and injury particulars. Shift work was defined as any work schedule that deviates from the hours of 7 am and 3 pm. Shift work may encompass evening, night, and early morning shifts, as well as fixed or rotating schedules.^14^

The Job was categorized into 4 groups based on the type of exposure. For example, the office workers, sellers, or guardians of the organization were categorized as an administrative group.

Manual workers included carpenters, plumbers, welders, etc. Construction workers were excluded as a separate group because of the high frequency of their injuries in previous Iranian studies. 

B. The Epworth Sleepiness Scale (ESS) questionnaire to evaluate the sleepiness of the patient; and trauma severity obtained from patient’s medical records.

The ESS is a self-administered questionnaire with 8 questions. Respondents are asked to rate, on a 4-point scale (0-3), their usual chances of dozing off or falling asleep while engaged in eight different activities. A score of 10 and higher is considered sleepy.^15^ In this study, the Persian version of the questionnaire was used which was previously translated and authenticated by Sadeghniiat.^16^

A. To evaluate injury severity in workers, injury severity score (ISS) criteria was used, which is one of the most common trauma scoring systems. ISS is a mathematical model for estimating injury severity. ISS is based on abbreviated injury scale (AIS) criteria, which measures the severity of anatomical and tissue injury. The lesion AIS number varies from 1 to 6 in the form of 1(Minor), 2 (Moderate), 3 (Serious but not life-threatening), 4 (Severe and life-threatening), 5 (Critical), 6 (Unsurvivable). The patient's lesions are divided into 6 areas of the body, including the head and neck, face, chest, abdomen and pelvic contents, limbs, and superficial parts of the body. To calculate the ISS, three regions with the most AIS are selected based on them. Finally, the severity of trauma was categorized into two categories based on the ISS: mild (ISS<8) and severe trauma (ISS>8).^17^

To perform descriptive analysis of qualitative variables, frequency distribution and for quantitative variables, mean and standard deviation were used. To determine the relationship between the severity of trauma and demographic factors, the ANOVA statistical test was used, and to determine the relationship between two qualitative variables, the Chi-square and Fisher exact tests were used. Data was analyzed by SPSS 20.0 

## Results

**Figure 1:**
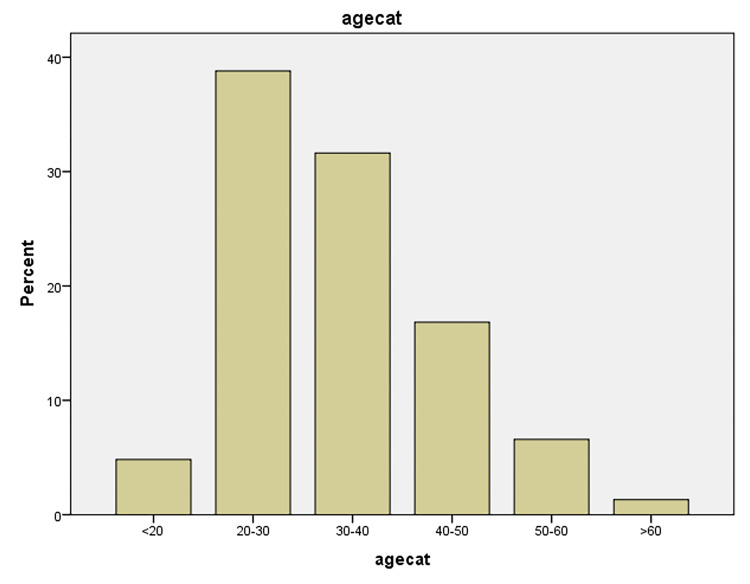
Age range of patients participating in the study

A total of 704 patients were referred to the emergency room of four teaching hospitals in Mashhad with occupational trauma. There were 21 participants with incomplete questionnaires and after removing them, a total of 683 patients’ data were analyzed. The mean (±SD) age and work experience of the participants were 34 (±10) and 10 (±9), respectively. Most of the participants were in the age group of 20-40 years, which includes young and middle-aged workers. (Figure 1) Other demographic characteristics of the participants are shown in Table 1 . The most patients were men, under bachelor education levels, manual workers, and had a shift work schedule.

**Table 1:** demographic information of the patients.

Variable		N (%)
**Gender**	male	636(93)
	female	47(7)
**Marital status **	Single	139(20)
	Married	543(80)
**Occupation**	Manual Worker	532(78)
	Administrative job	80(12)
	construction worker	24(4)
	Other	47(7)
**Education **	Under bachelor degree	631(92)
	Bachelor's degree and higher	52(8)
**Smoking **	Yes	204(30)
	No	479(70)
**shiftwork**	Yes	455(67)
	No	229(33)
**Sleepiness**	Yes	154(22)
	No	550(78)
**Opioid user **	Yes	98(14)
	No	584(86)
**Job **	construction worker	24(3%)
	Manual Worker	532(78%)
	Administrative job	80(12%)
	others	47(7%)

* Differences was evaluated using Pearson’s chi-square and one-way ANOVA test.

Table 2 represents the characteristics of trauma in participants. As seen in the table, the most common mechanism of trauma was falling. (%46)

**Table 2:** Trauma characteristic of the patients.

Variable		n (%)
**Mechanism of accident**	Fall	314(46)
	Cut	216(32)
	Explosion	6(1)
	Electric	10(2)
	Others	137(20)
**Location of accident**	Workplace	527(77)
	Outside the workplace	156(23)
**Daytime of accident**	Morning	359 (53%)
	Afternoon	196 (29%)
	Evening	77(12%)
	Night	51(7%)

According to Table 3, there was not a significant association between trauma severity and demographic characteristics. Those who had falling or electric accidents had more severe trauma compared to other injured patients. (p=0.00) 

**Table 3:** The correlation between trauma severity and demographic or accident characteristics.

		Mild	Moderate	Severe	p-value
**Gender **	Male	363(59%)	202(33%)	46 (8%)	0.9
	Female	26(59%)	14(32%)	4(9%)	
Age Mean(±SD)		34.4±9	34.2±10	34.2±10	0.9
**Marital status **	Single	82(61%)	44(33%)	8(6%)	0.5
	Married	307(59%)	171(33%)	42(8%)	
**Smoking**	No	275(60%)	149(33%)	35(8%)	0.91
	Yes	114(58%)	67(34%)	15(8%)	
**Drug use**	No	338(61%)	180(32%)	41(7%)	0.5
	Yes	51(53%)	36(38%)	9(9)	
**Education**	Under bachelor	361(60%)	198(33%)	46(8%)	0.8
	Bachelor and higher	28(56%)	18(36%)	4(8%)	
**Sleepiness**	Yes	91(55%)	63(38%)	12(7%)	0.29
	No	298(61%)	153(31%)	38(8%)	
**shiftwork**	Yes	265(61%)	142(33%)	31(7%)	0.6
	No	123(57%)	73(34%)	19(9%)	
**Mechanism of accident**	Fall	182(60 %)	88(29%)	32(11%)	0.00
	Cut	113(55%)	85(42%)	6(3%)	
	Explosion	2(40%)	3(60%)	0	
	Electric	2(20%)	7(70%)	1(10%)	
	Others	90(67 %)	33(25%)	11(8%)	

**Figure 2:**
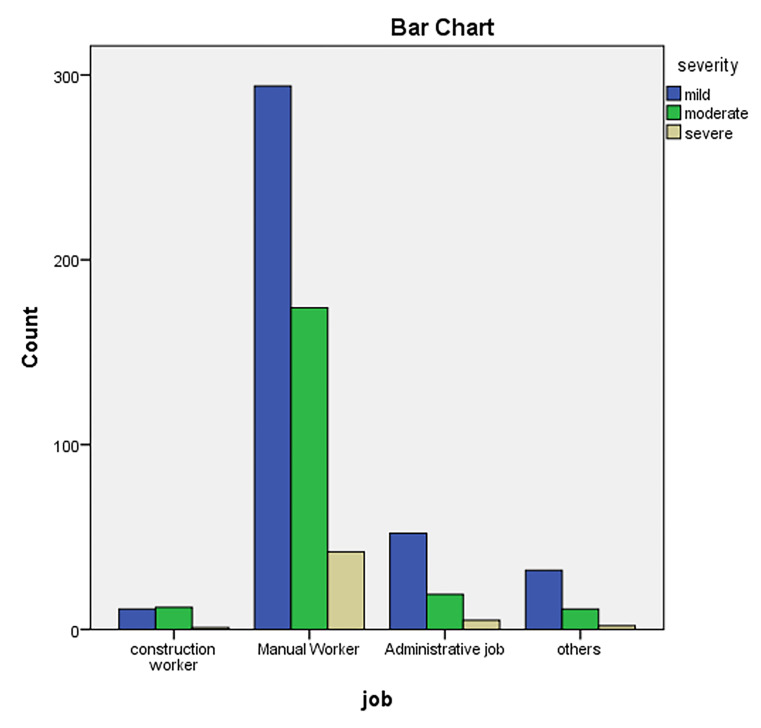
Trauma severity among different job categories.

Figure 2 demonstrates injury severity among job categories of injured patients. As seen, the most injury frequency was among manual working. Moreover, manual workers had the most severe injury proportion (compared to other workers). The detailed percentages are shown in Table 4. 

**Table 4:** The correlation between trauma severity and occupational characteristics.

		Trauma severity			p-value
		Mild	Moderate	Severe	
		N(%)					
Occupation	Manual job	294(58)	174(34)	42(8)	0.01
	Administrative job	52(68)	19(25)	5(7)	
	construction job	11(46)	12(50)	1(4)	
	Others	32(71)	11(24)	2(5)	
Work experience	10-20	118(20.1)	0	18(23.1)	0.46
	>20	63(10.7)	0	10(12.8)	
Shift work	Yes	265(61%)	142(32%)	31(7%)	0.6
	No	123(57%)	73(34%)	19(9%)	

In addition, the clinical course of the patients was followed. In total, among the patients included in the study, about 33% of the patients needed treatment without the need for follow-up. 28% of patients, needed follow-up in addition to treatment. Also, 84 patients, which includes 13% of the patients, needed to be hospitalized in the intensive care unit, and 171 patients needed to be hospitalized in other departments, which included about 26% of the patients. 580 patients needed paraclinical investigations such as imaging, which included more than 80% of patients. Table 5 shows the information related to the involvement of different body parts in the injured people of the present study. As shown in the table, the most injured limb is the hand followed by the leg.

**Table 5:** The percentage of injured body parts among patients.

Damaged member	n (%)
Head	92(14%)
Neck	14(2%)
Hand	241(35%)
Finger	73(11%)
Waist	17(3%)
Leg	144(21%)
other	98(14%)

## Discussion

The present study was conducted on 683 patients in four training centers in Mashhad for one year. In general, more than 90% of the patients were men. The average age of the participants in this study was 34.30 ± 10.51. Also, his average work experience was 10.18 ± 9.51. In the current study, 92% of injured patients had an education level under a bachelor’s degree and most of them (67%) were shift workers. The most frequent mechanism of accidents was fall (46%) and more than half of the accidents happened in the morning. Manual workers included 79% of injured patients. Most of the injuries (92%) were mild to moderate and only about 8 percent of patients had severe injuries. Also, most of these accidents took place in the morning work shifts.

In a study by Aghilinejad 10032 severe work-related injuries were gathered from the accident registry system of the governmental Iranian Mining and Mineral Industries Development & Renovation Organization (IMIDRO) between 2003 and 2011. 

The mean age and work experience of workers were 33. 9 ± (8. 4) years and 7. 7 (± 6. 6) years respectively. The most common accident mechanism was trauma and impact (27%) followed by falls (24. 1%). (18) These findings are in parallel with our results which show OI among young workers is more frequent.

Gholamizadeh and colleagues investigated occupational accidents in Iran statistics for 10 years (2007-2017) based on the Social Security Organization and reported that 98% of the OI patients were men. The findings showed that falls and slips (18.3%) and physical strikes (14.6%) were the most common causes of accidents. Accidents had the highest incidence rate from 9 AM to noon (34.77%). These findings are similar to our study findings which showed the most frequent injuries were falling and most accidents occurred in the morning.^19^

All registered OIs in the Bureau of Labor and Social Affairs of Kermanshah from 2009–2013 were analyzed in a cross-sectional study. Results indicated that most of the accidents occurred between 10 am and 1 pm, and the lowest number of accidents happened between 4 and 7 am. The most frequent accidents were related to the construction industry occurring. The main causes of the accidents were falls from the height.^20^ It is largely similar to our findings and like some Iranian studies, falls are one of the main causes of occupational accidents. In addition, the morning shift is a more common time for the occurrence of OI in several mentioned studies. 

Several studies similar to ours have reported the frequency of occupational accidents at younger ages. In a study conducted on evaluating safety and occupational health in open pit mines in Turkey, 18-24 and 24-30 years’ age groups were responsible for 55 percent of total accidents. Workers under 40 years old, were at a greater risk for occurrence of accidents compared to elder workers.^21^ In Vietnam also most of the occupational accidents took place in young people with under diploma education.^22^

The results of this study revealed a correlation between trauma severity and the mechanism of accident. (P value=0.00) We noticed no significant association between demographic features and severity of injury.

In our study, workers with less work experience had more severe traumas, but it was not significantly higher than more experienced workers. (p=0.4) Previous research on OI showed that young adults (under 30 years old) are at greater risk of accidents, maybe because of a lack of knowledge and work experience.^18^ Less physical strength and disabilities in older workers mean that there is a greater risk for them in jobs that need more strength or more expertise.^23^ On the other hand, older age is usually associated with more work experience, where more experience and disability of old age play an opposite role in the occurrence of accidents.^18,24^

Among the enrolled patients in the study, nearly a third of them (30%) required medical care without follow-up. An additional 222 patients, comprising 33% of the participants, necessitated follow-up in conjunction with medical care. Of the total number of patients, 84 (12.6%) necessitated hospitalization in the intensive care unit, while 171 patients (roughly 30%) required hospitalization in other wards.

In general, hand injuries included 35 percent of injuries, and after that, leg injuries, and head and finger injuries were the most common anatomical regions of injury. A study conducted by Izadi and colleagues in 2016 on a sample of 1000 injured workers revealed results similar to the present finding. According to their research, the most frequent causes and types of accidents were incaution, lack of attention, collision, and trapping, respectively. The limbs were observed to be the most commonly affected body parts. Furthermore, the study concluded that less than 1% of the reported occupational accidents resulted in fatalities. These findings provide crucial insight into the nature of workplace accidents and the associated risks, which can help guide effective preventive measures.^25^

**Limitations and Strengths**

There were some limitations in our study including the non-cooperation of some patients due to the acute conditions that happened to them. Also, the researcher couldn't be present in more than one medical center at the same time.

One of the strengths of this study was its multicenter nature. Also, since in this study, the relevant information was completed by the emergency medicine researcher; therefore, there is a high degree of confidence in the accuracy of the estimated injury severity and other required information.

## Conclusion

In general, the highest frequency of occupational accidents and the most severe injuries were related to men and manual workers. The reason can be attributed to the lack of use of safety devices. Most of them happened due to falls and cuts. Most of the injured people were working shifts and most of the injuries happened in the morning shift. Young age and less work experience were associated with more severe injuries. 

The results of this study showed the need for serious policies to reduce occupational injuries. The victims were the active workforce of society and at young ages who could work for many years. In addition to imposing treatment costs and absence from work on the health economy, this issue has financial and psychological consequences for the individual and his family.

Moreover, hand injuries (as the most common injured limb), minimized the possibility of continuing work in the short and medium term for the injured worker.

Strictness in complying with laws related to occupational health and safety, optimization of the work shift schedule to increase alertness, and necessary training for workers and employers are among the methods of reducing occupational accidents.

**Acknowledgement**

 The authors are grateful to all the individuals who participated in the present study.

## References

[1] Tehran T, Mohammad F, Vahedian-Shahroodi M (2016). A review of studies in the field of knowledge and safe behaviors of workers in Iran. Journal of Health Literacy.

[2] Azadeh-Fard N, Schuh A, Rashedi E, Camelio JA (2015). Risk assessment of occupational injuries using Accident Severity Grade. Safety Science.

[3] Leigh J, Macaskill P, Kuosma E, Mandryk J (1999). Global burden of disease and injury due to occupational factors. Epidemiology.

[4] Varacallo M, Knoblauch DK. Occupational Injuries and Workers' Compensation Management Strategies. Treasure Island (FL): StatPearls Publishing, 2024 Jan, https://www.ncbi.nlm.nih.gov/books/NBK470372/, accessed 4 February 2024. 29262238

[5] Ghods K, Shahinfar H, Razavi M, Mirmohammadkhani M, Pahlevan D (2016). Assessment the occupational accidents and its related factors in an industrial pipe factory:A case - control study in Iran. Koomesh Journal of Semnan University of Medical Sciences.

[6] Cui Y, Tian S-S, Qiao N, Wang C, Wang T, Huang J-J (2015). Associations of Individual-Related and Job-Related Risk Factors with Nonfatal Occupational Injury in the Coal Workers of Shanxi Province: A Cross-Sectional Study. PLOS ONE.

[7] Santana VS, Xavier C, Moura MCP, Oliveira R, Espírito-Santo J-S (2009). Araújo G. Severity of occupational injuries treated in emergency services. Rev Saude Publica.

[8] Hämäläinen P, Takala J, Saarela KL (2006). Global estimates of occupational accidents. Safety Science.

[9] Fan W. Chinese Occupational Safety Situation, Gap and Counter measure. China Coal Industry Publishing House, 2003.

[10] Lu M-L, Nakata A, Park JB, Swanson NG (2014). Workplace psychosocial factors associated with work-related injury absence: a study from a nationally representative sample of Korean workers. Int J Behav Med.

[11] Lee H, Chae D, Yi KH, Im S, Cho SH (2015). Multiple risk factors for work-related injuries and illnesses in Korean-Chinese migrant workers. Workplace Health Saf.

[12] Molineri A, Signorini ML, Tarabla HD (2015). Risk factors for work-related injury among farm workers: a 1-year study. Rural Remote Health.

[13] Bakhtiyari M, Aghaie A, Delpisheh A, Akbarpour S, Zayeri F, Soori H (2012). An Epidemiologic Survey of Recorded Job-Related Accidents by Iranian Social Security Organization (2001-2005).. JRUMS.

[14] Redeker NS, Caruso CC, Hashmi SD, Mullington JM, Grandner M, Morgenthaler TI (2019). Workplace Interventions to Promote Sleep Health and an Alert, Healthy Workforce. J Clin Sleep Med.

[15] Johns MW (1991). A new method for measuring daytime sleepiness: the Epworth sleepiness scale. Sleep.

[16] Sadeghniiat Haghighi K, Montazeri A, Khajeh Mehrizi A, Aminian O, Rahimi Golkhandan A, Saraei M, Sedaghat M (2013). The Epworth Sleepiness Scale: translation and validation study of the Iranian version. Sleep Breath.

[17] Baker SP, O'Neill B, Haddon WJr, Long WB (1974). The injury severity score: a method for describing patients with multiple injuries and evaluating emergency care. J Trauma.

[18] Aghilinejad M, Kouhpayezade J, Noori MK, Golabadi M (2013). Association of age and work experience with work-related injuries in mining and mineral industries in Iran 2003-2011. Razi Journal of Medical Sciences.

[19] Gholamizadeh K, Tapak L, Mohammadfam I, Soltanzadeh A (2022). Investigating the Work-related Accidents in Iran: Analyzing and Comparing the Factors Associated with the Duration of Absence from Work. Iranian Rehabilitation Journal.

[20] Ghanbari M, Ashtarian H, Yarmohammadi H (2017). An investigation of the frequency of the occupational accident in Kermanshah, Iran (2009-2013). Annals of Tropical Medicine and Public Health.

[21] Ural S, Demirkol S (2008). Evaluation of occupational safety and health in surface mines. Safety Science.

[22] Phung DT, Nguyen HT, Mock C, Keifer M (2008). Occupational injuries reported in a population-based injury survey in Vietnam. Int J Occup Environ Health.

[23] Chau N, Wild P, Dehaene D, Benamghar L, Mur JM, Touron C (2010). Roles of age, length of service and job in work-related injury: a prospective study of 446 120 person-years in railway workers. Occup Environ Med.

[24] Castillo-Rosa J, Suárez-Cebador M, Rubio-Romero JC, Aguado JA (2017). Personal factors and consequences of electrical occupational accidents in the primary, secondary and tertiary sectors. Safety Science.

[25] Izadi N, Aminian O, Esmaeili B (2019). Occupational Accidents in Iran: Risk Factors and Long Term Trend (2007-2016).. J Res Health Sci.

